# Efficient Temporal Third/Fourth-Order Finite Element Method for a Time-Fractional Mobile/Immobile Transport Equation with Smooth and Nonsmooth Data

**DOI:** 10.3390/ma14195792

**Published:** 2021-10-03

**Authors:** Lijuan Nong, An Chen

**Affiliations:** 1 College of Science, Guilin University of Technology, Guilin 541004, China; ljnong2013@163.com; 2 Center for Data Analysis and Algorithm Technology, Guilin University of Technology, Guilin 541004, China

**Keywords:** Riemann–Liouville derivative, mobile/immobile transport equation, convolution quadrature, third/fourth-order backward difference formula, finite element method, error estimates

## Abstract

In recent years, the numerical theory of fractional models has received more and more attention from researchers, due to the broad and important applications in materials and mechanics, anomalous diffusion processes and other physical phenomena. In this paper, we propose two efficient finite element schemes based on convolution quadrature for solving the time-fractional mobile/immobile transport equation with the smooth and nonsmooth data. In order to deal with the weak singularity of solution near t=0, we choose suitable corrections for the derived schemes to restore the third/fourth-order accuracy in time. Error estimates of the two fully discrete schemes are presented with respect to data regularity. Numerical examples are given to illustrate the effectiveness of the schemes.

## 1. Introduction

In this paper, we are interested in the robust high-order numerical methods for the following time-fractional mobile/immobile transport equation [1]:

(1) $$\begin{array}{ccc} \left(\kappa_{1}\partial_{t}+\kappa_{2}{}_{RL}D_{0,t}^{\alpha }\right)\left(u\left(x,t\right)-v\right) & = & \mu \Delta u(x,t)+f(x,t),\;\mathrm{in}\;\Omega \;\times \;(0,T], \end{array}$$

which is subject to the boundary condition u=0 on ∂Ω×(0,T] and the initial condition u(x,0)=v(x) in $\bar{\Omega }$. Here, $\Omega \subset R^{2}$ is a bounded convex polygonal domain and the parameters $\kappa_{1},\kappa_{2}$, and μ are positive constants. The source term *f* and the initial condition *v* are both given functions. The notation ${}_{RL}D_{0,t}^{\alpha }$ with α∈(0,1) denotes the Riemann–Liouville derivative defined by

$${}_{RL}D_{0,t}^{\alpha }u\left(\cdot ,t\right)=\frac{1}{\Gamma (1-\alpha )}\frac{\partial }{\partial t}\int_{0}^{t}{\left(t-s\right)}^{-\alpha }u\left(\cdot ,s\right)ds.$$

In recent years, Equation (1) has received extensive attention, and it has significant applications in diverse fields, especially in the modeling of groundwater solutes [2,3,4]. There are some numerical studies for Equation (1) or its variants; see [1,5,6,7,8,9] and the references therein.

In our recent paper [1], we studied the solution regularity of Equation (1) and proposed two efficient finite element schemes by employing convolution quadrature based on the backward Euler and the second-order backward difference methods. It is proved that the proposed schemes are robust with respect to data regularity, including the nonsmooth initial data, i.e., $v\in L^{2}\left(\Omega \right)$. One of the distinct features of the two schemes is that they do not impose any compatibility conditions on the source term *f*, so this avoids some unrealistic assumption for the solution regularity, i.e., the assumption of the sufficiently smooth solution. When dealing with the nonsmooth solution problem, we observe numerically that the convolution quadrature generated by the high-order backward difference formula (denoted by BDF) is only of first-order accuracy when no corrections are added. This motivates us to investigate how to choose some suitable corrections in order to restore the desired high-order accuracy.

Since the convolution quadrature has a nice stable discrete structure, for which it is easy to perform the analysis of the resulting numerical scheme [10,11], it seems that it is suitable for developing high-order numerical schemes of the fractional model with the Riemann–Liouville derivative. In order to restore the high-order accuracy, one usually needs to add some corrections to the original scheme. There are a few papers on this direction [12,13,14,15,16,17,18], just to name a few. One may refer to two review papers [19,20] for more details. In [14,16,17], the convolution quadratures generated by the backward Euler and the second-order backward difference methods were applied to obtain the robust scheme for the time-fractional Cattaneo equation, the two-term time-fractional diffusion-wave equation, and the modified anomalous subdiffusion equation, respectively. In [12], the authors constructed and analyzed an efficient fractional Crank–Nicolson finite element scheme with two corrections at the starting time levels. They proved that the proposed scheme is of second-order accuracy in time for both smooth and nonsmooth data. Recently, Wang et al. improved the results by adding only one single correction at the starting time level and presented the optimal error estimates of their resulting scheme [18]. In [13], Jin et al. developed the BDF*k* (with the *k* denoting the order of BDF) convolution quadrature with suitable correction terms to deal with the temporal discretization of the fractional evolution equation. Later on, Shi and Chen extended the BDF*k* convolution quadrature to numerically solve the fractional Feynman–Kac equation with Lévy flight [15]. As far as we know, there is no convolution quadrature generated by high-order BDF to solve Equation (1) with smooth and nonsmooth problem data. The goal of this paper is to fill this gap.

The contributions of this paper are listed below. First, based on the convolution quadrature generated by BDF3/BDF4, we develop the robust temporal third/fourth-order finite element scheme for problem (1) by carefully choosing the suitable corrections at the starting two/three steps. Second, the error estimates of the resulting schemes are expressed in term of data regularity, and are proved rigorously, cf. Theorems 2–5. Third, the designed numerical tests show that the two schemes indeed yield the temporal third/fourth-order theoretical accuracy, so this further verifies numerically the correctness of the error estimates and illustrates the significant improvement in the temporal accuracy of our schemes compared to that of [1], cf. Tables 4–6 in Section 6.

The rest of this paper is organized as follows. In Section 2, we propose the temporal third-order finite element scheme based on the convolution quadrature generated by BDF3. In Section 3, the method to obtain the suitable correction terms is discussed in detail. In Section 4, we prove that the BDF3 is of third-order accuracy, with respect to smooth and nonsmooth data. The generalization of the BDF3 is given in Section 5, and numerical examples are provided in Section 6 to verify the effectiveness of the two proposed numerical schemes. Finally, we present the conclusion in the last section, i.e., Section 7. The symbol *c* in this paper denotes a positive constant that is independent of the temporal and spatial step sizes.

## 2. The Temporal Third-Order Numerical Scheme

In this section, we propose a temporal third-order fully discrete scheme for Equation (1) based on convolution quadrature in time and the standard Galerkin finite element method in space.

### 2.1. Spatial Semidiscrete Scheme

We first introduce the semidiscrete scheme by using the finite element method in space and recall the corresponding error estimates [1].

Denote the continuous piecewise linear finite element space $V_{h}$ as follows:

$$V_{h}=\left\{v_{h}\in H_{0}^{1}\left(\Omega \right):v_{h}{|}_{K}\;\mathrm{is}\;\mathrm{the}\;\mathrm{linear}\;\mathrm{function}\;\mathrm{and}\;\forall K\in T_{h}\right\},$$

where $T_{h}$ is the partition of the domain Ω with $h=\max_{K\in T_{h}}h_{K}$, and $h_{K}$ is the diameter of the triangles *K* [21]. The semidiscrete scheme for (1) is given by the following: Find $u_{h}\left(t\right)=u_{h}\left(\cdot ,t\right)\in V_{h}$ such that the following holds:

$$\left(\left(\kappa_{1}\partial_{t}+\kappa_{2}{}_{RL}D_{0,t}^{\alpha }\right)\left(u_{h}-v_{h}\right),\chi \right)+\mu \left(\nabla u_{h},\nabla \chi \right)=\left(f,\chi \right),\;\forall \chi \in V_{h},$$

with the initial value condition $u_{h}\left(0\right)=v_{h}\in V_{h}$. Here, $v_{h}$ is the proper approximation to the function *v*. In this paper, we choose $v_{h}=P_{h}v$ for $v\in L^{2}\left(\Omega \right)$ and $v_{h}=R_{h}v$ when $v\in D\left(\Delta \right)=H_{0}^{1}\left(\Omega \right)\cap H^{2}\left(\Omega \right)$ [21]. Here, $P_{h}$ and $R_{h}$ are the $L^{2}\left(\Omega \right)$ orthogonal projection operator and Ritz projection operator, respectively. By using the discrete Laplacian $-\Delta_{h}:V_{h}\to V_{h}$, defined by

$$-\left(\Delta_{h}\varphi ,\chi \right)=\left(\nabla \varphi ,\nabla \chi \right),\;\forall \varphi ,\chi \in V_{h},$$

one further has

(2) $$\left(\kappa_{1}\partial_{t}+\kappa_{2}{}_{RL}D_{0,t}^{\alpha }\right)\left(u_{h}\left(t\right)-v_{h}\right)-\mu \Delta_{h}u_{h}\left(t\right)=f_{h}\left(t\right),\;t>0,$$

where $f_{h}\left(t\right)=P_{h}f\left(t\right)$.

The error estimates for the semidiscrete scheme (2) were investigated in [1]; we collect the corresponding results as follows.

**Theorem** **1.** *Denote the space $S:=L^{\infty }\left(0,T;L^{2}\left(\Omega \right)\right)$. Then, for the semidiscrete scheme *(2)*, we have*

*(a)*  *If $v\in L^{2}\left(\Omega \right)$, then $∥u\left(t\right)-u_{h}\left(t\right)∥\leq ch^{2}(t^{-\alpha }∥v∥+|\ln\left(t^{\alpha }/h^{2}\right){|∥f∥}_{S}).$*
*(b)*  *If v∈D(Δ), then $∥u\left(t\right)-u_{h}\left(t\right)∥\leq ch^{2}(∥\Delta v∥+|\ln\left(t^{\alpha }/h^{2}\right){|∥f∥}_{S}).$*

### 2.2. Fully Discrete Schemes

We divide the time interval [0,T] into uniform grids ${\left\{t_{n}=n\tau \right\}}_{n=0}^{N}$ with τ=T/N. Here, *N* is a given positive integer. Based on the convolution quadrature generated by BDF*k*, the numerical approximation of ${}_{RL}D_{0,t}^{\alpha }u_{h}\left(t\right)$ at $t=t_{n}$ is described by

$$\partial_{\tau }^{\alpha }u_{h}^{n}=\frac{1}{\tau^{\alpha }}\sum_{j=0}^{n}w_{n-j}u_{h}^{j},$$

in which $u_{h}^{n}=u_{h}\left(t_{n}\right)$ and the weights $w_{j}$ are provided by

(3) $$\frac{1}{\tau^{\alpha }}\sum_{j=0}^{\infty }w_{j}\xi^{j}=\delta_{\tau }{\left(\xi \right)}^{\alpha },$$

with $\delta_{\tau }\left(\xi \right)=\frac{1}{\tau }\sum_{j=1}^{k}\frac{1}{j}{\left(1-\xi \right)}^{j}$.

Applying the convolution quadrature generated by BDF3/BDF4 for solving the semidiscrete scheme (2), one may obtain the following fully discrete finite element scheme: Find the numerical approximation $U_{h}^{n},n\geq 1$ of $u(t_{n})$, such that the following holds:

(4) $$\left(\kappa_{1}\partial_{\tau }+\kappa_{2}\partial_{\tau }^{\alpha }\right)\left(U_{h}^{n}-v_{h}\right)-\mu \Delta_{h}U_{h}^{n}=F_{h}^{n},$$

where $n=1,2,\cdots ,N,U_{h}^{0}=v_{h}$, and $F_{h}^{n}=f_{h}\left(t_{n}\right)$. It is known that the scheme (4) generally exhibits only temporal first-order accuracy, due to the low regularity of the solution near t=0. Such a fact is also verified by the numerical examples in Section 6.

In what follows of this section, we consider the scheme (4) generated by BDF3 and use the idea presented in [13] to derive a modified scheme which can restore the temporal third-order accuracy. To this end, we define ${\tilde{f}}_{h}\left(t\right)=f_{h}\left(t\right)-f_{h}\left(0\right)-tf_{h}^{\prime }\left(0\right)$ and denote $w_{h}\left(t\right)=u_{h}\left(t\right)-v$. The semidiscrete scheme (2) can be rewritten as follows:

(5) $$\left(\kappa_{1}\partial_{t}+\kappa_{2}{}_{RL}D_{0,t}^{\alpha }\right)w_{h}\left(t\right)-\mu \Delta_{h}w_{h}\left(t\right)=\mu \Delta_{h}v_{h}+f_{h}\left(0\right)+tf_{h}^{\prime }\left(0\right)+{\tilde{f}}_{h}\left(t\right).$$

In view of the construction of BDF2 in [1], we may naturally consider the following two corrections for (4) at the starting two steps:

(6) $$\left(\kappa_{1}\partial_{\tau }+\kappa_{2}\partial_{\tau }^{\alpha }\right)W_{h}^{1}-\mu \Delta_{h}W_{h}^{1}=\lambda_{11}\left(\mu \Delta_{h}v_{h}+f_{h}\left(0\right)\right)+\lambda_{12}\tau f_{h}^{\prime }\left(0\right)+{\tilde{f}}_{h}^{1},$$

and

(7) $$\left(\kappa_{1}\partial_{\tau }+\kappa_{2}\partial_{\tau }^{\alpha }\right)W_{h}^{2}-\mu \Delta_{h}W_{h}^{2}=\lambda_{21}\left(\mu \Delta_{h}v_{h}+f_{h}\left(0\right)\right)+2\lambda_{22}\tau f_{h}^{\prime }\left(0\right)+{\tilde{f}}_{h}^{2},$$

where $W_{h}^{n}=U_{h}^{n}-v,n\geq 0$. The four parameters $\lambda_{ij}\left(i,j=1,2\right)$ are unknown and need to be determined.

## 3. Determination of the Two Corrections

In this part, we determine the four parameters $\lambda_{ij}\left(i,j=1,2\right)$ in the two corrections (6) and (7) based on the comparison of the semidiscrete solution in (5) and the fully discrete solution in (4). Let the notation $\hat{\left(\cdot \right)}$ be the Laplace transform of (·), i.e., $\hat{g}\left(z\right):=\int_{0}^{\infty }e^{-zt}g\left(t\right)dt$. Denote the kernel K(z) as follows:

(8) $$K\left(z\right)=z^{-1}{\left(\kappa_{1}z+\kappa_{2}z^{\alpha }-\mu \Delta_{h}\right)}^{-1}.$$

Define the generating function $\bar{g}\left(\xi \right)=\sum_{n=0}^{\infty }g^{n}\xi^{n}$ with a given sequence ${\left(g^{n}\right)}_{n=0}^{\infty }$. We have the following results for the integral representations.

**Lemma** **1.** *The semidiscrete solution in* (5) *and the fully discrete solution in* (4) *(with $W_{h}^{n}$ replacing $U_{h}^{n}$) have the following integral representations:*

(9) $$w_{h}\left(t\right)=\frac{1}{2\pi i}\int_{\Gamma_{\theta ,\delta }}e^{zt}\left(K\left(z\right)\left(\mu \Delta_{h}v_{h}+f_{h}\left(0\right)\right)+z^{-1}K\left(z\right)f_{h}^{\prime }\left(0\right)+zK\left(z\right){\hat{\tilde{f}}}_{h}\left(z\right)\right)dz,$$

*and*

(10) $$\begin{array}{ccc} W_{h}^{n} & = & \frac{1}{2\pi i}\int_{\Gamma_{\theta ,\delta }^{\tau }}e^{zt_{n}}K\left(\delta_{\tau }\left(e^{-z\tau }\right)\right)(\tau \delta_{\tau }\left(e^{-z\tau }\right)\varpi_{1}\left(e^{-z\tau }\right)\left(\mu \Delta_{h}v_{h}+f_{h}\left(0\right)\right)\\ & & +\delta_{\tau }\left(e^{-z\tau }\right)\varpi_{2}\left(e^{-z\tau }\right)\tau^{2}f_{h}^{\prime }\left(0\right)+\tau \delta_{\tau }\left(e^{-z\tau }\right){\bar{\tilde{F}}}_{h}\left(e^{-z\tau }\right))dz, \end{array}$$

*where*

$$\varpi_{1}\left(z\right)=\frac{z}{1-z}+\left(\lambda_{11}-1\right)z+\left(\lambda_{12}-1\right)z^{2},$$

*and*

$$\varpi_{2}\left(z\right)=\frac{z}{{\left(1-z\right)}^{2}}+\left(\lambda_{12}-1\right)z+\left(2\lambda_{22}-1\right)z^{2}.$$

*Here, the contours $\Gamma_{\theta ,\delta }:=\left\{re^{\pm i\theta }:r\geq \delta \right\}\cup \{\delta e^{i\phi }:|\phi |\leq \theta \}$ with δ>0 and θ∈(π/2,π) and Γθ,δτ:={z∈Γθ,δ:|Im(z)|≤π/τ}.*

**Proof.**  By using the Laplace transform technology, we can easily obtain the integral representation (9) from (5). One may refer to [1] for further details.

It remains to derive the fully discrete solution in (4) based on discrete Laplace transform, i.e., generating function.

Let ${\tilde{F}}_{h}^{n}=F_{h}^{n}-f_{h}^{0}-t_{n}f_{h}^{\prime }\left(0\right)$. The scheme (4) has the following equivalent form:

(11) $$\left(\kappa_{1}\partial_{\tau }+\kappa_{2}\partial_{\tau }^{\alpha }\right)W_{h}^{n}-\mu \Delta_{h}W_{h}^{n}=\mu \Delta_{h}v_{h}+f_{h}\left(0\right)+t_{n}f_{h}^{\prime }\left(0\right)+{\tilde{F}}_{h}^{n},\;n\geq 3,$$

with the two initial corrections (6) and (7). Multiplying $\xi^{n}$ on both sides of (11), and summing up for n=1,2,⋯, we obtain the following:

(12) $$\begin{array}{ccc} & & \sum_{n=1}^{\infty }\left(\kappa_{1}\partial_{\tau }+\kappa_{2}\partial_{\tau }^{\alpha }\right)W_{h}^{n}\xi^{n}-\mu \sum_{n=1}^{\infty }\Delta_{h}W_{h}^{n}\xi^{n}\\ & = & \left(\sum_{n=3}^{\infty }\xi^{n}+\lambda_{11}\xi +\lambda_{12}\xi^{2}\right)\left(\mu \Delta_{h}v_{h}+f_{h}\left(0\right)\right)\\ & & +\left(\sum_{n=3}^{\infty }n\xi^{n}+\lambda_{12}\xi +2\lambda_{22}\xi^{2}\right)\tau f_{h}^{\prime }\left(0\right)+{\bar{\tilde{F}}}_{h}\left(\xi \right). \end{array}$$

Noting $W_{h}^{0}=0$, one has

$$\sum_{n=1}^{\infty }\left(\kappa_{1}\partial_{\tau }+\kappa_{2}\partial_{\tau }^{\alpha }\right)W_{h}^{n}\xi^{n}=\left(\kappa_{1}\delta_{\tau }\left(\xi \right)+\kappa_{2}\delta_{\tau }{\left(\xi \right)}^{\alpha }\right){\bar{W}}_{h}\left(\xi \right).$$

From (12), we have

(13) $$\begin{array}{ccc} & & \left(\kappa_{1}\delta_{\tau }\left(\xi \right)+\kappa_{2}\delta_{\tau }{\left(\xi \right)}^{\alpha }-\mu \Delta_{h}\right){\bar{W}}_{h}\left(\xi \right)\\ & = & \left(\frac{\xi }{1-\xi }+\left(\lambda_{11}-1\right)\xi +\left(\lambda_{12}-1\right)\xi^{2}\right)\left(\mu \Delta_{h}v_{h}+f_{h}\left(0\right)\right)\\ & & +\left(\frac{\xi }{{\left(1-\xi \right)}^{2}}+\left(\lambda_{12}-1\right)\xi +\left(2\lambda_{22}-1\right)\xi^{2}\right)\tau f_{h}^{\prime }\left(0\right)+{\bar{\tilde{F}}}_{h}\left(\xi \right). \end{array}$$

Thus,

$$\begin{array}{ccc} {\bar{W}}_{h}\left(\xi \right) & = & {\left(\kappa_{1}\delta_{\tau }\left(\xi \right)+\kappa_{2}\delta_{\tau }{\left(\xi \right)}^{\alpha }-\mu \Delta_{h}\right)}^{-1}\\ & & \cdot (\left(\frac{\xi }{1-\xi }+\left(\lambda_{11}-1\right)\xi +\left(\lambda_{12}-1\right)\xi^{2}\right)\left(\mu \Delta_{h}v_{h}+f_{h}\left(0\right)\right)\\ & & +\left(\frac{\xi }{{\left(1-\xi \right)}^{2}}+\left(\lambda_{12}-1\right)\xi +\left(2\lambda_{22}-1\right)\xi^{2}\right)\tau f_{h}^{\prime }\left(0\right)+{\bar{\tilde{F}}}_{h}\left(\xi \right)), \end{array}$$

from which we have

(14) $$W_{h}^{n}=\frac{1}{2\pi i}\int_{|\xi |=r}\xi^{-n-1}{\bar{W}}_{h}\left(\xi \right)d\xi =\frac{\tau }{2\pi i}\int_{\Gamma_{r}^{\tau }}e^{zt_{n}}{\bar{W}}_{h}\left(e^{-z\tau }\right)dz,$$

with r∈(0,1) and the notation $\Gamma_{r}^{\tau }:=\left\{z=-\ln\left(r\right)/\tau +iy:y\in R\;\mathrm{and}\;|y|\leq \pi /\tau \right\}$. Here, we have used the change of variables $\xi =e^{-z\tau }$ in the second equality of (14). Denote the complex region as Σ enclosed by $\Gamma_{r}^{\tau },\Gamma_{\theta ,\delta }^{\tau }$, and $\Gamma_{\pm }^{\tau }$, where $\Gamma_{\pm }^{\tau }:=R\pm i\pi /\tau$. Note that the integrand $e^{zt_{n}}{\bar{W}}_{h}\left(e^{-z\tau }\right)$ in (14) is analytic with respect to *z* in Σ, so applying the Cauchy’s theorem, we have

$$\frac{\tau }{2\pi i}\left(\int_{\Gamma_{r}^{\tau }}-\int_{\Gamma_{+}^{\tau }}-\int_{\Gamma_{\theta ,\delta }^{\tau }}+\int_{\Gamma_{-}^{\tau }}\right)e^{zt_{n}}{\bar{W}}_{h}\left(e^{-z\tau }\right)dz=0.$$

This leads to the desired result (10) since there holds

$$\int_{\Gamma_{+}^{\tau }}e^{zt_{n}}{\bar{W}}_{h}\left(e^{-z\tau }\right)dz=\int_{\Gamma_{-}^{\tau }}e^{zt_{n}}{\bar{W}}_{h}\left(e^{-z\tau }\right)dz.$$

All this completes the proof of the lemma.  □

From the representations of (9) and (10), it is intuitive to select $\lambda_{ij}\left(i,j=1,2\right)$ in (10) such that

(15) $$∥K\left(\delta_{\tau }\left(e^{-z\tau }\right)\right)\delta_{\tau }\left(e^{-z\tau }\right)\varpi_{1}\left(e^{-z\tau }\right)\tau -K\left(z\right)∥\leq c\tau^{3},$$

and

(16) $$∥K\left(\delta_{\tau }\left(e^{-z\tau }\right)\right)\delta_{\tau }\left(e^{-z\tau }\right)\varpi_{2}\left(e^{-z\tau }\right)\tau^{2}-z^{-1}K\left(z\right)∥\leq c\tau^{3},$$

for any given $z\in \Gamma_{\theta ,\delta }^{\tau }$.

For the left hand sides of (15) and (16), the triangle inequality leads to

(17) $$\begin{array}{ccc} & & ∥\left(\delta_{\tau }\left(e^{-z\tau }\right)\varpi_{1}\left(e^{-z\tau }\right)\tau -1\right)K\left(\delta_{\tau }\left(e^{-z\tau }\right)\right)+K\left(\delta_{\tau }\left(e^{-z\tau }\right)\right)-K\left(z\right)∥\\ & \leq & |\delta_{\tau }\left(e^{-z\tau }\right)\varpi_{1}\left(e^{-z\tau }\right)\tau -1|∥K\left(\delta_{\tau }\left(e^{-z\tau }\right)\right)∥+∥K\left(\delta_{\tau }\left(e^{-z\tau }\right)\right)-K\left(z\right)∥, \end{array}$$

and

(18) $$\begin{array}{ccc} & & ∥{\left(\kappa_{1}\delta_{\tau }\left(e^{-z\tau }\right)+\kappa_{2}\delta_{\tau }{\left(e^{-z\tau }\right)}^{\alpha }-\mu \Delta_{h}\right)}^{-1}\left(\varpi_{2}\left(e^{-z\tau }\right)\tau^{2}-\delta_{\tau }{\left(e^{-z\tau }\right)}^{-2}\right)\\ & & +\delta_{\tau }{\left(e^{-z\tau }\right)}^{-1}K\left(\delta_{\tau }\left(e^{-z\tau }\right)\right)-z^{-1}K\left(z\right)∥\\ & \leq & ∥{\left(\kappa_{1}\delta_{\tau }\left(e^{-z\tau }\right)+\kappa_{2}\delta_{\tau }{\left(e^{-z\tau }\right)}^{\alpha }-\mu \Delta_{h}\right)}^{-1}∥|\varpi_{2}\left(e^{-z\tau }\right)\tau^{2}-\delta_{\tau }{\left(e^{-z\tau }\right)}^{-2}|\\ & & +∥\delta_{\tau }{\left(e^{-z\tau }\right)}^{-1}K\left(\delta_{\tau }\left(e^{-z\tau }\right)\right)-z^{-1}K\left(z\right)∥. \end{array}$$

We shall need the following lemma.

**Lemma** **2.** *Let K(z) and $\delta_{\tau }\left(\xi \right)$ be defined as in *(8)* and *(3)* with k=3, respectively. Then, for any $z\in \Gamma_{\theta ,\delta }^{\tau }$, we have*

$$∥K\left(\delta_{\tau }\left(e^{-z\tau }\right)\right)-K\left(z\right)∥\leq c\tau^{3}{\left|z\right|}^{2-\alpha }.$$

**Proof.**  Since $K\left(z\right)=z^{-1}{\left(\kappa_{1}z+\kappa_{2}z^{\alpha }-\mu \Delta_{h}\right)}^{-1}$, we have

$$\begin{array}{ccc} & & ∥K\left(\delta_{\tau }\left(e^{-z\tau }\right)\right)-K\left(z\right)∥\\ & \leq & ∥\delta_{\tau }{\left(e^{-z\tau }\right)}^{-1}{\left(\kappa_{1}\delta_{\tau }\left(e^{-z\tau }\right)+\kappa_{2}\delta_{\tau }{\left(e^{-z\tau }\right)}^{\alpha }-\mu \Delta_{h}\right)}^{-1}-z^{-1}{\left(\kappa_{1}z+\kappa_{2}z^{\alpha }-\mu \Delta_{h}\right)}^{-1}∥\\ & \leq & |\delta_{\tau }{\left(e^{-z\tau }\right)}^{-1}-z^{-1}|∥{\left(\kappa_{1}\delta_{\tau }\left(e^{-z\tau }\right)+\kappa_{2}\delta_{\tau }{\left(e^{-z\tau }\right)}^{\alpha }-\mu \Delta_{h}\right)}^{-1}∥\\ & & +{\left|z\right|}^{-1}∥{\left(\kappa_{1}\delta_{\tau }\left(e^{-z\tau }\right)+\kappa_{2}\delta_{\tau }{\left(e^{-z\tau }\right)}^{\alpha }-\mu \Delta_{h}\right)}^{-1}-{\left(\kappa_{1}z+\kappa_{2}z^{\alpha }-\mu \Delta_{h}\right)}^{-1}∥. \end{array}$$

In view of Lemma B.1. in [13], we derive that

(19) $$\begin{array}{ccc} |\delta_{\tau }{\left(e^{-z\tau }\right)}^{-1}-z^{-1}| & \leq & c\tau^{3}{\left|z\right|}^{2},\\ ∥{\left(\kappa_{1}\delta_{\tau }\left(e^{-z\tau }\right)+\kappa_{2}\delta_{\tau }{\left(e^{-z\tau }\right)}^{\alpha }-\mu \Delta_{h}\right)}^{-1}∥ & \leq & c\min\{|\delta_{\tau }\left(e^{-z\tau }\right){|}^{-1},|\delta_{\tau }\left(e^{-z\tau }\right){|}^{-\alpha }\} \end{array}$$

(20) $$\begin{array}{ccc} & \leq & c\min\{|z{|}^{-1},|z{|}^{-\alpha }\}, \end{array}$$

and

$$\begin{array}{ccc} & & ∥{\left(\kappa_{1}\delta_{\tau }\left(e^{-z\tau }\right)+\kappa_{2}\delta_{\tau }{\left(e^{-z\tau }\right)}^{\alpha }-\mu \Delta_{h}\right)}^{-1}-{\left(\kappa_{1}z+\kappa_{2}z^{\alpha }-\mu \Delta_{h}\right)}^{-1}∥\\ & \leq & |\kappa_{1}z+\kappa_{2}z^{\alpha }-\kappa_{1}\delta_{\tau }\left(e^{-z\tau }\right)-\kappa_{2}\delta_{\tau }{\left(e^{-z\tau }\right)}^{\alpha }|\\ & & \cdot ∥{\left(\kappa_{1}\delta_{\tau }\left(e^{-z\tau }\right)+\kappa_{2}\delta_{\tau }{\left(e^{-z\tau }\right)}^{\alpha }-\mu \Delta_{h}\right)}^{-1}∥∥{\left(\kappa_{1}z+\kappa_{2}z^{\alpha }-\mu \Delta_{h}\right)}^{-1}∥\\ & \leq & c\tau^{3}{\left(|z\right|}^{4}+{\left|z\right|}^{3+\alpha }{)\cdot \min\{|z|}^{-1}{,|z|}^{-\alpha }\}. \end{array}$$

Thus, we further deduce that

$$\begin{array}{ccc} & & ∥K\left(\delta_{\tau }\left(e^{-z\tau }\right)\right)-K\left(z\right)∥\\ & \leq & c\tau^{3}{\left|z\right|}^{2}{\min\{|z|}^{-1}{,|z|}^{-\alpha }\}+c\tau^{3}{\left(|z\right|}^{3}+{\left|z\right|}^{2+\alpha }{)\cdot \min\{|z|}^{-1}{,|z|}^{-\alpha }\}\\ & \leq & c\tau^{3}{\left(|z\right|}^{2}+{\left|z\right|}^{3}+{\left|z\right|}^{2+\alpha }{)\min\{|z|}^{-1}{,|z|}^{-\alpha }\}\leq c\tau^{3}{\left|z\right|}^{2-\alpha }, \end{array}$$

for $z\in \Gamma_{\theta ,\delta }^{\tau }$, where the last inequality holds since |z|<1.  □

By Lemma 2, we only need to choose

(21) $$|\delta_{\tau }\left(e^{-z\tau }\right)\varpi_{1}\left(e^{-z\tau }\right){\tau -1|\leq c|z|}^{3}\tau^{3},$$

and

(22) $$|\varpi_{2}\left(e^{-z\tau }\right)\tau^{2}-\delta_{\tau }{\left(e^{-z\tau }\right)}^{-2}\left|\leq c|z\right|\tau^{3}.$$

It is derived in ([13], cf. (16) and (17)) that the above error estimates can be valid by choosing $\lambda_{11}=23/12,\lambda_{12}=13/12,\lambda_{21}=7/12$, and $\lambda_{22}=1/2$. So, the suitable corrections in (6) and (7) can be written as follows:

(23) $$\left(\kappa_{1}\partial_{\tau }+\kappa_{2}\partial_{\tau }^{\alpha }\right)U_{h}^{1}-\mu \Delta_{h}U_{h}^{1}=11/12\left(\mu \Delta_{h}v_{h}+f_{h}\left(0\right)\right)+1/12\tau f_{h}^{\prime }\left(0\right)+F_{h}^{1},$$

and

(24) $$\left(\kappa_{1}\partial_{\tau }+\kappa_{2}\partial_{\tau }^{\alpha }\right)U_{h}^{2}-\mu \Delta_{h}U_{h}^{2}=-5/12\left(\mu \Delta_{h}v_{h}+f_{h}\left(0\right)\right)+F_{h}^{2}.$$

The fully discrete scheme

(25) $$\left(\kappa_{1}\partial_{\tau }+\kappa_{2}\partial_{\tau }^{\alpha }\right)U_{h}^{n}-\mu \Delta_{h}U_{h}^{n}=F_{h}^{n},\;n\geq 3,$$

with the two corrections (23) and (24) as the starting two steps is referred to as the BDF3, and can restore third-order accuracy in time, which will be proved in the next section.

## 4. Error Estimates of the BDF3

In this part, we present the error estimates for the modified scheme (25). We first consider the error estimate for a homogenous problem with v∈D(Δ) and f=0. In the proof of error estimates, we may explicitly or implicitly employ the identity $\Delta_{h}R_{h}=P_{h}\Delta$ and the $L^{2}$-stability of the orthogonal projection $P_{h}$ when needed.

**Theorem** **2.** *Let u and $U_{h}^{n}$ be the solutions of *(1)* and *(25)*, respectively. Assuming v∈D(Δ) and f=0. Then, for $v_{h}:=R_{h}v$, we have*

$$∥u\left(t_{n}\right)-U_{h}^{n}∥\leq c\left(h^{2}+\tau^{3}t_{n}^{\alpha -3}\right)∥\Delta v∥.$$

**Proof.**  It suffices to consider the bound of $∥u_{h}\left(t_{n}\right)-U_{h}^{n}∥$ in view of Theorem 1. From (9) and (10) in Lemma 1, we can obtain

(26) $$\begin{array}{ccc} ∥w_{h}\left(t_{n}\right)-W_{h}^{n}∥ & \leq & ∥\frac{1}{2\pi i}\int_{\Gamma_{\theta ,\delta }^{\tau }}e^{zt_{n}}\left(K\left(z\right)-K\left(\delta_{\tau }\left(e^{-z\tau }\right)\right)\tau \delta_{\tau }\left(e^{-z\tau }\right)\varpi_{1}\left(e^{-z\tau }\right)\right)\left(\mu \Delta_{h}v_{h}\right)dz∥\\ & & +∥\frac{1}{2\pi i}\int_{\Gamma_{\theta ,\delta }\setminus \Gamma_{\theta ,\delta }^{\tau }}e^{zt_{n}}K\left(z\right)\left(\mu \Delta_{h}v_{h}\right)dz∥=:I_{1}+I_{2}. \end{array}$$

Since $∥K\left(\delta_{\tau }\left(e^{-z\tau }\right)\right){∥\leq c\min\{|z|}^{-1}{,|z|}^{-\alpha }\}$, in view of (17) with Lemma 2 and letting $\delta \leq 1/t_{n}$, we have

$$\begin{array}{ccc} I_{1} & \leq & c\tau^{3}∥\Delta_{h}v_{h}∥\left(\int_{\delta }^{\pi /(\tau \sin\theta )}e^{rt_{n}\cos\theta }r^{2-\alpha }dr+\int_{-\theta }^{\theta }e^{\delta t_{n}\left|\cos\eta \right|}\delta^{3-\alpha }d\eta \right)\\ & \leq & c\tau^{3}t_{n}^{\alpha -3}∥\Delta v∥. \end{array}$$

For $I_{2}$, we get

$$I_{2}\leq c∥\Delta_{h}v_{h}∥\int_{\pi /(\tau \sin\theta )}^{\infty }e^{t_{n}r\cos\theta }r^{-\alpha -1}dr.$$

Note that r≥π/(τsinθ), one has $r^{-1}\leq r^{2}\tau^{3}{\left(\sin\theta /\pi \right)}^{3}$. So, we have

$$\begin{array}{ccc} I_{2} & \leq & c\tau^{3}∥\Delta_{h}v_{h}∥\int_{\pi /(\tau \sin\theta )}^{\infty }e^{t_{n}r\cos\theta }r^{2-\alpha }dr\\ & \leq & ct_{n}^{\alpha -3}\tau^{3}∥\Delta_{h}v_{h}∥\int_{0}^{\infty }e^{s\cos\theta }s^{2-\alpha }ds\\ & \leq & ct_{n}^{\alpha -3}\tau^{3}∥\Delta v∥. \end{array}$$

Putting the error estimates of the two parts, $I_{1}$ and $I_{2}$, into (26), we complete the proof of the theorem. □

Next, we state the error estimate for the homogenous problem with $v\in L^{2}\left(\Omega \right)$ and f=0.

**Theorem** **3.** *Let u and $U_{h}^{n}$ be the solutions of *(1)* and *(25)*, respectively. Assuming $v\in L^{2}\left(\Omega \right)$ and f=0. Then, for $v_{h}:=P_{h}v$ we have*

$$∥u\left(t_{n}\right)-U_{h}^{n}∥\leq c\left(h^{2}t_{n}^{-\alpha }+\tau^{3}t_{n}^{-3}\right)∥v∥.$$

**Proof.**  Let $\tilde{K}\left(z\right)=\mu {\left(\kappa_{1}z+\kappa_{1}z^{\alpha }-\mu \Delta_{h}\right)}^{-1}\Delta_{h}$. In view of (26), we have

$$\begin{array}{ccc} & & ∥w_{h}\left(t_{n}\right)-W_{h}^{n}∥\\ & \leq & ∥\frac{1}{2\pi i}\int_{\Gamma_{\theta ,\delta }^{\tau }}e^{zt_{n}}\tilde{\tilde{K}}\left(z\right)v_{h}dz∥\\ & & +∥\frac{1}{2\pi i}\int_{\Gamma_{\theta ,\delta }\setminus \Gamma_{\theta ,\delta }^{\tau }}e^{zt_{n}}z^{-1}\tilde{K}\left(z\right)v_{h}dz∥=:I_{1}+I_{2}, \end{array}$$

where $\tilde{\tilde{K}}\left(z\right)=z^{-1}\tilde{K}\left(z\right)-\tau \varpi_{1}\left(e^{-z\tau }\right)\tilde{K}\left(\delta_{\tau }\left(e^{-z\tau }\right)\right)$. By the triangle inequality, the term $\tilde{\tilde{K}}\left(z\right)$ has the following error estimate:

$$∥\tilde{\tilde{K}}\left(z\right)∥\leq |\tau \varpi_{1}\left(e^{-z\tau }\right)-z^{-1}|∥\tilde{K}\left(\delta_{\tau }\left(e^{-z\tau }\right)\right){∥+|z|}^{-1}∥\tilde{K}\left(\delta_{\tau }\left(e^{-z\tau }\right)\right)-\tilde{K}\left(z\right)∥=:I_{11}+I_{12}.$$

For the first term $I_{11}$, using the result in (19) and the criterion (21), we have

$$\begin{array}{ccc} & & |\tau \varpi_{1}\left(e^{-z\tau }\right)-z^{-1}|\\ & \leq & |\tau \varpi_{1}\left(e^{-z\tau }\right)\delta_{\tau }\left(e^{-z\tau }\right)\left(\delta_{\tau }{\left(e^{-z\tau }\right)}^{-1}-z^{-1}\right)|\\ & & +|\left(\tau \varpi_{1}\left(e^{-z\tau }\right)\delta_{\tau }\left(e^{-z\tau }\right)-1\right){\left||z\right|}^{-1}\leq c\tau^{3}{\left|z\right|}^{2}. \end{array}$$

So,

$$\begin{array}{ccc} I_{11} & \leq & c\tau^{3}{\left|z\right|}^{2}∥\tilde{K}\left(\delta_{\tau }\left(e^{-z\tau }\right)\right)∥\\ & \leq & c\tau^{3}{\left|z\right|}^{2}∥{\left(\kappa_{1}\delta_{\tau }\left(e^{-z\tau }\right)+\kappa_{2}\delta_{\tau }{\left(e^{-z\tau }\right)}^{\alpha }-\mu \Delta_{h}\right)}^{-1}\left(\kappa_{1}\delta_{\tau }\left(e^{-z\tau }\right)+\kappa_{2}\delta_{\tau }{\left(e^{-z\tau }\right)}^{\alpha }\right)-I∥\\ & \leq & c\tau^{3}{\left|z\right|}^{2}. \end{array}$$

For the second term $I_{12}$, we have

$$\begin{array}{ccc} \left|z\right|I_{12} & = & ∥\left(\kappa_{1}\delta_{\tau }\left(e^{-z\tau }\right)+\kappa_{2}\delta_{\tau }{\left(e^{-z\tau }\right)}^{\alpha }\right){\left(\kappa_{1}\delta_{\tau }\left(e^{-z\tau }\right)+\kappa_{2}\delta_{\tau }{\left(e^{-z\tau }\right)}^{\alpha }-\mu \Delta_{h}\right)}^{-1}\\ & & -\left(\kappa_{1}z+\kappa_{2}z^{\alpha }\right){\left(\kappa_{1}z+\kappa_{2}z^{\alpha }-\mu \Delta_{h}\right)}^{-1}∥\\ & \leq & ∥\left[\kappa_{1}\delta_{\tau }\left(e^{-z\tau }\right)+\kappa_{2}\delta_{\tau }{\left(e^{-z\tau }\right)}^{\alpha }-\left(\kappa_{1}z+\kappa_{2}z^{\alpha }\right)\right]{\left(\kappa_{1}\delta_{\tau }\left(e^{-z\tau }\right)+\kappa_{2}\delta_{\tau }{\left(e^{-z\tau }\right)}^{\alpha }-\mu \Delta_{h}\right)}^{-1}∥\\ & & +∥\left(\kappa_{1}z+\kappa_{2}z^{\alpha }\right)\left[{\left(\kappa_{1}\delta_{\tau }\left(e^{-z\tau }\right)+\kappa_{2}\delta_{\tau }{\left(e^{-z\tau }\right)}^{\alpha }-\mu \Delta_{h}\right)}^{-1}-{\left(\kappa_{1}z+\kappa_{2}z^{\alpha }-\mu \Delta_{h}\right)}^{-1}\right]∥\\ & =: & I_{121}+I_{122}. \end{array}$$

Applying the error estimates in Lemma B.1. of [13] again, we derive that

$$I_{121}\leq c\tau^{3}{\left(|z\right|}^{4}+{\left|z\right|}^{3+\alpha }{)\min\{|z|}^{-1}{,|z|}^{-\alpha }\}\leq c\tau^{3}{\left|z\right|}^{3}.$$

Since

$$\begin{array}{ccc} I_{122} & = & ∥\left(\kappa_{1}z+\kappa_{2}z^{\alpha }\right)\left[{\left(\kappa_{1}\delta_{\tau }\left(e^{-z\tau }\right)+\kappa_{2}\delta_{\tau }{\left(e^{-z\tau }\right)}^{\alpha }-\mu \Delta_{h}\right)}^{-1}-{\left(\kappa_{1}z+\kappa_{2}z^{\alpha }-\mu \Delta_{h}\right)}^{-1}\right]∥\\ & \leq & ∥\left(\kappa_{1}z+\kappa_{2}z^{\alpha }\right)\left[\kappa_{1}\delta_{\tau }\left(e^{-z\tau }\right)+\kappa_{2}\delta_{\tau }{\left(e^{-z\tau }\right)}^{\alpha }-\left(\kappa_{1}z+\kappa_{2}z^{\alpha }\right)\right]\\ & & \cdot {\left(\kappa_{1}\delta_{\tau }\left(e^{-z\tau }\right)+\kappa_{2}\delta_{\tau }{\left(e^{-z\tau }\right)}^{\alpha }-\mu \Delta_{h}\right)}^{-1}{\left(\kappa_{1}z+\kappa_{2}z^{\alpha }-\mu \Delta_{h}\right)}^{-1}∥\\ & = & |\kappa_{1}z+\kappa_{2}z^{\alpha }|∥{\left(\kappa_{1}z+\kappa_{2}z^{\alpha }-\mu \Delta_{h}\right)}^{-1}∥\cdot I_{121}\leq c\tau^{3}{\left|z\right|}^{3}, \end{array}$$

we obtain the error estimate for $I_{12}$: $I_{12}\leq c\tau^{3}{\left|z\right|}^{2}.$

Consequently, by choosing $\delta \leq 1/t_{n}$, we obtain

$$\begin{array}{ccc} I_{1} & \leq & c\tau^{3}∥v_{h}∥\left(\int_{\delta }^{\pi /(\tau \sin\theta )}e^{rt_{n}\cos\theta }r^{2}dr+\int_{-\theta }^{\theta }e^{\delta t_{n}\left|\cos\eta \right|}\delta^{3}d\eta \right)\\ & \leq & ct_{n}^{-3}\tau^{3}∥v∥. \end{array}$$

For the second term $I_{2}$, similar to the derivation of $I_{2}$ in Theorem 2, we can easily derive the following inequality:

$$\begin{array}{ccc} I_{2} & \leq & c∥v_{h}∥\int_{\pi /(\tau \sin\theta )}^{\infty }e^{rt_{n}\cos\theta }r^{-1}dr\\ & \leq & c\tau^{3}∥v_{h}∥\int_{0}^{\infty }e^{s\cos\theta }s^{2}ds\leq ct_{n}^{-3}\tau^{3}∥v∥, \end{array}$$

where the first inequality is valid since $∥\tilde{K}\left(z\right)∥=∥\left(\kappa_{1}z+\kappa_{2}z^{\alpha }\right){\left(\kappa_{1}z+\kappa_{2}z^{\alpha }-\mu \Delta_{h}\right)}^{-1}-I∥\leq c$. This, together with the error estimate of $I_{1}$ and Theorem 1, ends the proof. □

Now, we consider the inhomogenous problem, i.e., f≠0 and v=0.

**Theorem** **4.** *Let u and $U_{h}^{n}$ be the solutions of *(1)* and *(25)* with v=0, respectively. Then, we have*

$$\begin{array}{ccc} ∥u\left(t_{n}\right)-U_{h}^{n}∥ & \leq & c(h^{2}|\ln\left(t_{n}^{\alpha }/h^{2}\right){|∥f∥}_{S}+\tau^{3}t_{n}^{\alpha -3}∥f\left(0\right)∥\\ & & +\tau^{3}t_{n}^{\alpha -2}∥f^{\prime }\left(0\right)∥+\tau^{3}t_{n}^{\alpha -1}∥f^{\prime \prime }\left(0\right)∥+\tau^{3}\int_{0}^{t_{n}}{\left(t_{n}-s\right)}^{\alpha -1}∥f^{\prime \prime \prime }\left(s\right)∥ds). \end{array}$$

**Proof.**  It follows from (9) and (10) in Lemma 1 that

$$∥w_{h}\left(t_{n}\right)-W_{h}^{n}∥\leq I_{1}+I_{2}+I_{3},$$

where

$$\begin{array}{ccc} I_{1} & = & ∥\frac{1}{2\pi i}\int_{\Gamma_{\theta ,\delta }^{\tau }}e^{zt_{n}}\left(K\left(z\right)-K\left(\delta_{\tau }\left(e^{-z\tau }\right)\right)\tau \delta_{\tau }\left(e^{-z\tau }\right)\varpi_{1}\left(e^{-z\tau }\right)\right)f_{h}\left(0\right)dz∥\\ & & +∥\frac{1}{2\pi i}\int_{\Gamma_{\theta ,\delta }\setminus \Gamma_{\theta ,\delta }^{\tau }}e^{zt_{n}}K\left(z\right)f_{h}\left(0\right)dz∥,\\ I_{2} & = & ∥\frac{1}{2\pi i}\int_{\Gamma_{\theta ,\delta }^{\tau }}e^{zt_{n}}\left(z^{-1}K\left(z\right)-K\left(\delta_{\tau }\left(e^{-z\tau }\right)\right)\delta_{\tau }\left(e^{-z\tau }\right)\varpi_{2}\left(e^{-z\tau }\right)\tau^{2}\right)f_{h}^{\prime }\left(0\right)dz∥\\ & & +∥\frac{1}{2\pi i}\int_{\Gamma_{\theta ,\delta }\setminus \Gamma_{\theta ,\delta }^{\tau }}e^{zt_{n}}K\left(z\right)z^{-1}f_{h}^{\prime }\left(0\right)dz∥=:I_{21}+I_{22},\\ I_{3} & = & ∥\frac{1}{2\pi i}\int_{\Gamma_{\theta ,\delta }}e^{zt_{n}}zK\left(z\right){\hat{\tilde{f}}}_{h}\left(z\right)dz-\frac{1}{2\pi i}\int_{\Gamma_{\theta ,\delta }^{\tau }}e^{zt_{n}}K\left(\delta_{\tau }\left(e^{-z\tau }\right)\right)\tau \delta_{\tau }\left(e^{-z\tau }\right){\bar{\tilde{F}}}_{h}\left(e^{-z\tau }\right)dz∥. \end{array}$$

From the derivation in Theorem 2, we can easily obtain the following error estimate of $I_{1}$:

$$I_{1}\leq c\tau^{3}t_{n}^{\alpha -3}∥f\left(0\right)∥.$$

Next, we consider the two terms $I_{2}$ and $I_{3}$, respectively. Through the triangle inequality, we can obtain

$$\begin{array}{ccc} & & ∥z^{-1}K\left(z\right)-K\left(\delta_{\tau }\left(e^{-z\tau }\right)\right)\delta_{\tau }\left(e^{-z\tau }\right)\varpi_{2}\left(e^{-z\tau }\right)\tau^{2}∥\\ & \leq & ∥{\left(\kappa_{1}\delta_{\tau }\left(e^{-z\tau }\right)+\kappa_{2}\delta_{\tau }{\left(e^{-z\tau }\right)}^{\alpha }-\mu \Delta_{h}\right)}^{-1}\left[\delta_{\tau }{\left(e^{-z\tau }\right)}^{-2}-\varpi_{2}\left(e^{-z\tau }\right)\tau^{2}\right]∥\\ & & +∥z^{-2}{\left(\kappa_{1}z+\kappa_{2}z^{\alpha }-\mu \Delta_{h}\right)}^{-1}-\delta_{\tau }{\left(e^{-z\tau }\right)}^{-2}{\left(\kappa_{1}\delta_{\tau }\left(e^{-z\tau }\right)+\kappa_{2}\delta_{\tau }{\left(e^{-z\tau }\right)}^{\alpha }-\mu \Delta_{h}\right)}^{-1}∥\\ & =: & I_{21}+I_{22}. \end{array}$$

Since $∥{\left(\kappa_{1}\delta_{\tau }\left(e^{-z\tau }\right)+\kappa_{2}\delta_{\tau }{\left(e^{-z\tau }\right)}^{\alpha }-\mu \Delta_{h}\right)}^{-1}{∥\leq c\min\{|z|}^{-1}{,|z|}^{-\alpha }\}$, using the criterion (16), we have $I_{21}\leq c\tau^{3}{\left|z\right|}^{1-\alpha }$. For $I_{22}$, the following inequality holds:

$$\begin{array}{ccc} I_{22} & \leq & ∥\delta_{\tau }{\left(e^{-z\tau }\right)}^{-2}\left[{\left(\kappa_{1}z+\kappa_{2}z^{\alpha }-\mu \Delta_{h}\right)}^{-1}-{\left(\kappa_{1}\delta_{\tau }\left(e^{-z\tau }\right)+\kappa_{2}\delta_{\tau }{\left(e^{-z\tau }\right)}^{\alpha }-\mu \Delta_{h}\right)}^{-1}\right]∥\\ & & +∥\left(z^{-2}-\delta_{\tau }{\left(e^{-z\tau }\right)}^{-2}\right){\left(\kappa_{1}z+\kappa_{2}z^{\alpha }-\mu \Delta_{h}\right)}^{-1}∥. \end{array}$$

Analogous to the derivation of the error estimates of $I_{121}$ and $I_{122}$ for $I_{12}$ in Theorem 3, we can obtain $I_{22}\leq c\tau^{3}{\left|z\right|}^{1-\alpha }$. So, we choose $\delta =1/t_{n}$ and get

$$I_{2}\leq c\tau^{3}∥f_{h}^{\prime }\left(0\right)∥\left[\int_{\delta }^{\pi /(\tau \sin\theta )}e^{rt_{n}\cos\theta }r^{1-\alpha }dr+\int_{-\theta }^{\theta }e^{\delta t_{n}\left|\cos\eta \right|}\delta^{2-\alpha }d\eta \right]\leq c\tau^{3}t_{n}^{\alpha -2}∥f^{\prime }\left(0\right)∥.$$

For the last term $I_{3}$, by using the Taylor expansion of $f_{h}\left(t\right)$ at t=0, one has $f_{h}\left(t\right)=f_{h}\left(0\right)+tf_{h}^{\prime }\left(0\right)+\frac{t^{2}}{2}f_{h}^{\prime \prime }\left(0\right)+\frac{t^{2}}{2}*f_{h}^{\prime \prime \prime }$. So ${\tilde{f}}_{h}\left(t\right)=\frac{t^{2}}{2}f_{h}^{\prime \prime }\left(0\right)+\frac{t^{2}}{2}*f_{h}^{\prime \prime \prime }=:f_{1}\left(t\right)+f_{2}\left(t\right)$. It suffices to analyze the error estimates for source terms of the form $f_{1}\left(t\right)$ and $f_{2}\left(t\right)$, respectively. Assume that ${\tilde{f}}_{h}\left(t\right)\equiv f_{1}\left(t\right)$. Then the term $I_{3}$ has the following form:

$$\begin{array}{ccc} I_{3} & = & ∥\frac{1}{2\pi i}\int_{\Gamma_{\theta ,\delta }}e^{zt_{n}}z^{-2}K\left(z\right)f_{h}^{\prime \prime }\left(0\right)dz\\ & & -\frac{1}{2\pi i}\int_{\Gamma_{\theta ,\delta }^{\tau }}e^{zt_{n}}K\left(\delta_{\tau }\left(e^{-z\tau }\right)\right)\tau^{3}\varpi_{21}\left(\delta_{\tau }\left(e^{-z\tau }\right)\right)\delta_{\tau }\left(e^{-z\tau }\right)f_{h}^{\prime \prime }\left(0\right)dz∥, \end{array}$$

where $\varpi_{21}\left(\xi \right)=\xi \left(1+\xi \right)/{\left(1-\xi \right)}^{3}$. By repeating the similar argument in the above error estimate for $I_{2}$, one can derive that $I_{3}\leq c\tau^{3}t_{n}^{\alpha -1}∥f^{\prime \prime }\left(0\right)∥$. When ${\tilde{f}}_{h}\left(t\right)\equiv f_{2}\left(t\right)$, the corresponding semidiscrete Galerkin solution in (9) can be written as follows:

$$u_{h}\left(t_{n}\right)=\left(E*{\tilde{f}}_{h}\right)\left(t_{n}\right)=\left(E*\left(\frac{t^{2}}{2}*f_{h}^{\prime \prime \prime }\right)\right)\left(t_{n}\right)=\left(\left(E*\frac{t^{2}}{2}\right)*f_{h}^{\prime \prime \prime }\right)\left(t_{n}\right),$$

where the operator E(t) is given by

$$E\left(t\right)=\frac{1}{2\pi i}\int_{\Gamma_{\theta ,\delta }}e^{zt}{\left(\kappa_{1}z+\kappa_{2}z^{\alpha }-\mu \Delta_{h}\right)}^{-1}dz.$$

Furthermore, by (13), we have

$${\bar{U}}_{h}\left(\xi \right)={\left(\kappa_{1}\delta_{\tau }\left(\xi \right)+\kappa_{2}\delta_{\tau }{\left(\xi \right)}^{\alpha }-\mu \Delta_{h}\right)}^{-1}{\bar{f}}_{2}\left(\xi \right)=:\bar{E}\left(\delta_{\tau }\left(\xi \right)\right){\bar{f}}_{2}\left(\xi \right),$$

from which we can derive the representation of the fully discrete solution $U_{h}^{n}$ below:

(27) $$U_{h}^{n}=\sum_{k=0}^{n}E_{\tau }^{n-k}f_{2}\left(t_{k}\right),$$

with $\bar{E}\left(\delta_{\tau }\left(\xi \right)\right)=\sum_{n=0}^{\infty }E_{\tau }^{n}\xi^{n}$. In view of (14), we get

$$E_{\tau }^{n}=\frac{\tau }{2\pi i}\int_{\Gamma_{\theta ,\delta }^{\tau }}e^{zt_{n}}{\left(\kappa_{1}\delta_{\tau }\left(e^{-z\tau }\right)+\kappa_{2}\delta_{\tau }{\left(e^{-z\tau }\right)}^{\alpha }-\mu \Delta_{h}\right)}^{-1}dz.$$

Denote $E_{\tau }\left(t\right)=\sum_{n=0}^{\infty }E_{\tau }^{n}\delta_{t_{n}}\left(t\right)$ with the Dirac delta function $\delta_{t_{n}}\left(t\right)$ at $t_{n}$, we can rewrite (27) as

$$U_{h}^{n}=\left(E_{\tau }*f_{2}\right)\left(t_{n}\right)=\left(\left(E_{\tau }*\frac{t^{2}}{2}\right)*f_{h}^{\prime \prime \prime }\right)\left(t_{n}\right).$$

Since

$$\bar{E_{\tau }*\frac{t^{2}}{2}}\left(\xi \right)=\sum_{n=0}^{\infty }\sum_{j=0}^{n}E_{\tau }^{n-j}\frac{t_{j}^{2}}{2}\xi^{n}=\bar{E}\left(\delta_{\tau }\left(\xi \right)\right)\omega_{21}\left(\xi \right)\tau^{2},$$

one can derive that

$$∥\left(\left(E_{\tau }-E\right)*\frac{t^{2}}{2}\right)\left(t_{n}\right)∥\leq c\tau^{3}t_{n}^{\alpha -1}.$$

We show that the above inequality is valid for $\forall t\in (t_{n-1},t_{n})$. Indeed, by using the Taylor series expansion of E(t) at $t=t_{n}$, we have

$$\left(E*\frac{t^{2}}{2}\right)\left(t\right)=\left(E*\frac{t^{2}}{2}\right)\left(t_{n}\right)+\left(t-t_{n}\right)\left(E*t\right)\left(t_{n}\right)+\frac{{\left(t-t_{n}\right)}^{2}}{2}\left(E*1\right)\left(t_{n}\right)+\frac{1}{2}\int_{t_{n}}^{t}{\left(t-s\right)}^{2}E\left(s\right)ds.$$

This Taylor expansion also holds for $\left(E_{\tau }*\frac{t^{2}}{2}\right)\left(t\right)$. One can easily obtain

$$∥\left(\left(E_{\tau }-E\right)*t\right)\left(t_{n}\right)∥\leq c\tau^{2}t_{n}^{\alpha -1},$$

and

$$∥\left(\left(E_{\tau }-E\right)*1\right)\left(t_{n}\right)∥\leq c\tau t_{n}^{\alpha -1}.$$

Besides, there holds the following estimates:

$$∥\int_{t_{n}}^{t}\left(t-s\right)E\left(s\right)ds∥\leq c\int_{t_{n}}^{t}{\left(t-s\right)}^{2}s^{\alpha -1}ds∥\leq c\tau^{3}t^{\alpha -1},$$

and

$$∥\int_{t_{n}}^{t}\left(t-s\right)E_{\tau }\left(s\right)ds∥\leq c{\left(t_{n}-t\right)}^{2}∥E_{\tau }^{n}∥\leq c\tau^{3}t_{n}^{\alpha -1}\leq c\tau^{3}t^{\alpha -1},$$

with $t\in (t_{n-1},t_{n})$. Thus, combining with Theorem 1, we complete the proof. □

## 5. Extending to the Temporal Fourth-Order Scheme

In this section, we consider a higher-order numerical scheme based on the idea discussed in derivation of BDF3. For sake of simplicity, we focus on the temporal fourth-order scheme. By performing the preceding argument, we introduce temporal fourth-order finite element scheme (denoted by BDF4) based on convolution quadrature generated by BDF4: Find $U_{h}^{n},n\geq 1$, such that the following holds:

(28) $$\left(\kappa_{1}\partial_{\tau }+\kappa_{2}\partial_{\tau }^{\alpha }\right)U_{h}^{n}-\mu \Delta_{h}U_{h}^{n}=F_{h}^{n},\;n\geq 4,$$

with the following three corrections:

$$\begin{array}{ccc} \left(\kappa_{1}\partial_{\tau }+\kappa_{2}\partial_{\tau }^{\alpha }\right)U_{h}^{1}-\mu \Delta_{h}U_{h}^{1} & = & 31/24\left(\mu \Delta_{h}v_{h}+f_{h}\left(0\right)\right)+1/6\tau f_{h}^{\prime }\left(0\right)+F_{h}^{1},\\ \left(\kappa_{1}\partial_{\tau }+\kappa_{2}\partial_{\tau }^{\alpha }\right)U_{h}^{2}-\mu \Delta_{h}U_{h}^{2} & = & -7/6\left(\mu \Delta_{h}v_{h}+f_{h}\left(0\right)\right)-1/12\tau f_{h}^{\prime }\left(0\right)+F_{h}^{2}, \end{array}$$

and

$$\left(\kappa_{1}\partial_{\tau }+\kappa_{2}\partial_{\tau }^{\alpha }\right)U_{h}^{3}-\mu \Delta_{h}U_{h}^{3}=3/8\left(\mu \Delta_{h}v_{h}+f_{h}\left(0\right)\right)+F_{h}^{3}.$$

The corresponding error estimates for BDF4 is described by the following theorem.

**Theorem** **5.** *Let u and $U_{h}^{n}$ be the solutions of *(1)* and *(28)*, respectively. Then, we have*

$$\begin{array}{ccc} ∥u\left(t_{n}\right)-U_{h}^{n}∥ & \leq & c(h^{2}|\ln\left(t_{n}^{\alpha }/h^{2}\right){|∥f∥}_{S}+\left(h^{2}t_{n}^{\beta -\alpha }+\tau^{4}t_{n}^{\beta -4}\right)∥\tilde{v}∥+\tau^{4}t_{n}^{\alpha -4}∥f\left(0\right)∥\\ & & +\tau^{4}t_{n}^{\alpha -3}∥f^{\prime }\left(0\right)∥+\tau^{4}t_{n}^{\alpha -2}∥f^{\prime \prime }\left(0\right)∥+\tau^{4}t_{n}^{\alpha -1}∥f^{\prime \prime \prime }\left(0\right)∥\\ & & +\tau^{4}\int_{0}^{t_{n}}{\left(t_{n}-s\right)}^{\alpha -1}∥f^{\prime \prime \prime \prime }\left(s\right)∥ds), \end{array}$$

*where β=0 and $\tilde{v}=v$ if $v\in L^{2}\left(\Omega \right)$ and β=α and $\tilde{v}=\Delta v$ if v∈D(Δ).*

**Proof.**  Similar to the idea discussed in the error estimates of Theorems 2–4, one can easily deduce the desired error estimate results for BDF4, and thus we omit the details here. □

## 6. Numerical Examples

Now, we perform the numerical examples to test the error estimates of BDF3 (25) and BDF4 (28). Let $\Omega ={\left(0,1\right)}^{2}$ and T=0.1. We consider the following three types of problem data:

(a) v(x,y)=xy(1−x)(1−y) and f=0;
(b) $v\left(x,y\right)=\chi_{\left(0,3/4]\;\times \;(0,1\right)}\left(x,y\right)$ and f=0;
(c) v=0 and $f=\left(1+t^{4.1}\right)\chi_{\left(0,3/4]\;\times \;(0,1\right)}\left(x,y\right)$.

Here, $\chi_{S}$ is denoted as the characteristic function of the set *S*.

We remark that the three types of data considered above cover all cases of error theories, and therefore this is sufficient for our numerical tests. Moreover, we focus on the temporal convergence rate since the spatial one is well understood. Since the analytical solution of equation with the above data is hard to obtain, we use reference solution instead and the reference solution is computed by BDF4 with fixing h=1/10 and τ=T/4096. The $L^{2}\left(\Omega \right)$ norm errors are measured by $∥u\left(t_{n}\right)-U_{h}^{n}∥$. The numerical results are demonstrated in Table 1, Table 2, Table 3, Table 4, Table 5 and Table 6. From these tables, we can see that when no correction terms are added to BDF3 and BDF4, the temporal convergence accuracy is only first-order accuracy for both (see Table 1, Table 2 and Table 3), while after adding suitable correction terms (cf. Table 4, Table 5 and Table 6), we observe the theoretical temporal third-/fourth-order accuracy, which numerically verifies the importance of adding correction terms and the correctness of our constructed correction terms in BDF3/BDF4.

In addition, we also numerically compare the BDF3 and BDF4 with the BDF2 proposed in [1]; see Table 4, Table 5 and Table 6. The numerical results further indicate the correctness of our error estimates presented in Theorems 2–5 and reveal that our schemes greatly improve the temporal convergence accuracy of the BDF2.

## 7. Conclusions

In this paper, we developed an efficient temporal third/fourth-order finite element scheme for model (1) with smooth and nonsmooth data. The schemes were obtained by using the Galerkin finite element method in space and the convolution quadrature generated by the BDF3 and BDF4 in time, so this is why we refer to the resulting schemes as BDF3 and BDF4, respectively. In order to restore the desired accuracy for both smooth and nonsmooth data, we carefully chose the suitable corrections for the BDF3 and BDF4 at the starting two/three steps. Error estimates of the two modified schemes were established, with respect to the data regularity. Finally, the numerical examples confirmed the robustness and accuracy of the proposed schemes. With the BDF3/BDF4 modified at the starting two/three steps, we greatly improved the convergence results presented in [1].

## Figures and Tables

**Table 1:** The temporal $L^{2}$ norm errors for case (a) with h=1/10, by original schemes.

Scheme	*N*	α=0.1		α=0.5		α=0.9	
		$L^{2}$ Error	Rate	$L^{2}$ Error	Rate	$L^{2}$ Error	Rate
	16	$2.52\;\times \;10^{-4}$	-	2.67$\;\times \;10^{-4}$	-	3.52$\;\times \;10^{-4}$	-
BDF3 (25)	32	1.26$\;\times \;10^{-4}$	1.00	1.33$\;\times \;10^{-4}$	1.00	1.76$\;\times \;10^{-4}$	1.00
	64	6.27$\;\times \;10^{-5}$	1.01	6.64$\;\times \;10^{-5}$	1.00	8.78$\;\times \;10^{-5}$	1.00
	128	3.13$\;\times \;10^{-5}$	1.00	3.32$\;\times \;10^{-5}$	1.00	4.38$\;\times \;10^{-5}$	1.00
	16	2.55$\;\times \;10^{-4}$	-	2.70$\;\times \;10^{-4}$	-	3.55$\;\times \;10^{-4}$	-
BDF4 (28)	32	1.26$\;\times \;10^{-4}$	1.02	1.34$\;\times \;10^{-4}$	1.01	1.76$\;\times \;10^{-4}$	1.01
	64	6.27$\;\times \;10^{-5}$	1.01	6.65$\;\times \;10^{-5}$	1.01	8.78$\;\times \;10^{-5}$	1.00
	128	3.13$\;\times \;10^{-5}$	1.00	3.32$\;\times \;10^{-5}$	1.00	4.38$\;\times \;10^{-5}$	1.00

**Table 2:** The temporal $L^{2}$ norm errors for case (b) with h=1/10, by original schemes.

Scheme	*N*	α=0.1		α=0.5		α=0.9	
		$L^{2}$ Error	Rate	$L^{2}$ Error	Rate	$L^{2}$ Error	Rate
	16	5.25$\;\times \;10^{-3}$	-	5.57$\;\times \;10^{-3}$	-	7.39$\;\times \;10^{-3}$	-
BDF3 (25)	32	2.62$\;\times \;10^{-3}$	1.00	2.78$\;\times \;10^{-3}$	1.00	3.69$\;\times \;10^{-3}$	1.00
	64	1.30$\;\times \;10^{-3}$	1.01	1.39$\;\times \;10^{-3}$	1.00	1.84$\;\times \;10^{-3}$	1.00
	128	6.51$\;\times \;10^{-4}$	1.00	6.91$\;\times \;10^{-4}$	1.00	9.19$\;\times \;10^{-4}$	1.00
	16	5.31$\;\times \;10^{-3}$	-	5.62$\;\times \;10^{-3}$	-	7.44$\;\times \;10^{-3}$	-
BDF4 (28)	32	2.63$\;\times \;10^{-3}$	1.02	2.78$\;\times \;10^{-3}$	1.01	3.70$\;\times \;10^{-3}$	1.01
	64	1.31$\;\times \;10^{-3}$	1.01	1.39$\;\times \;10^{-3}$	1.01	1.84$\;\times \;10^{-3}$	1.00
	128	6.51$\;\times \;10^{-4}$	1.00	6.91$\;\times \;10^{-4}$	1.00	9.19$\;\times \;10^{-4}$	1.00

**Table 3:** The temporal $L^{2}$ norm errors for case (c) with h=1/10, by original schemes.

Scheme	*N*	α=0.1		α=0.5		α=0.9	
		$L^{2}$ Error	Rate	$L^{2}$ Error	Rate	$L^{2}$ Error	Rate
	16	2.59$\;\times \;10^{-4}$	-	2.75$\;\times \;10^{-4}$	-	3.63$\;\times \;10^{-4}$	-
BDF3 (25)	32	1.29$\;\times \;10^{-4}$	1.00	1.37$\;\times \;10^{-4}$	1.00	1.81$\;\times \;10^{-4}$	1.00
	64	6.45$\;\times \;10^{-5}$	1.01	6.83$\;\times \;10^{-5}$	1.00	9.04$\;\times \;10^{-5}$	1.00
	128	3.22$\;\times \;10^{-5}$	1.00	3.41$\;\times \;10^{-5}$	1.00	4.51$\;\times \;10^{-5}$	1.00
	16	2.63$\;\times \;10^{-4}$	-	2.77$\;\times \;10^{-4}$	-	3.65$\;\times \;10^{-4}$	-
BDF4 (28)	32	1.30$\;\times \;10^{-4}$	1.02	1.37$\;\times \;10^{-4}$	1.01	1.81$\;\times \;10^{-4}$	1.01
	64	6.45$\;\times \;10^{-5}$	1.01	6.84$\;\times \;10^{-5}$	1.01	9.04$\;\times \;10^{-5}$	1.00
	128	3.22$\;\times \;10^{-5}$	1.00	3.41$\;\times \;10^{-5}$	1.00	4.51$\;\times \;10^{-5}$	1.00

**Table 4:** The temporal $L^{2}$ norm errors for case (a) with h=1/10, by modified schemes.

Scheme	*N*	α=0.1		α=0.5		α=0.9	
		$L^{2}$ Error	Rate	$L^{2}$ Error	Rate	$L^{2}$ Error	Rate
	16	$3.03\;\times \;10^{-5}$	-	2.34$\;\times \;10^{-5}$	-	8.34$\;\times \;10^{-6}$	-
BDF2 [1]	32	7.56$\;\times \;10^{-6}$	2.00	5.81$\;\times \;10^{-6}$	2.01	2.17$\;\times \;10^{-6}$	1.94
	64	1.89$\;\times \;10^{-6}$	2.00	1.45$\;\times \;10^{-6}$	2.00	5.52$\;\times \;10^{-7}$	1.98
	128	4.71$\;\times \;10^{-7}$	2.00	3.62$\;\times \;10^{-7}$	2.00	1.39$\;\times \;10^{-7}$	1.99
	16	1.21$\;\times \;10^{-6}$	-	1.10$\;\times \;10^{-6}$	-	3.15$\;\times \;10^{-7}$	-
BDF3 (25)	32	1.67$\;\times \;10^{-7}$	2.86	1.45$\;\times \;10^{-7}$	2.93	3.18$\;\times \;10^{-8}$	3.31
	64	2.17$\;\times \;10^{-8}$	2.94	1.85$\;\times \;10^{-8}$	2.97	3.55$\;\times \;10^{-9}$	3.16
	128	2.75$\;\times \;10^{-9}$	2.98	2.33$\;\times \;10^{-9}$	2.99	4.20$\;\times \;10^{-10}$	3.08
	16	2.32$\;\times \;10^{-7}$	-	1.34$\;\times \;10^{-7}$	-	1.46$\;\times \;10^{-7}$	-
BDF4 (28)	32	3.59$\;\times \;10^{-9}$	6.02	2.00$\;\times \;10^{-9}$	6.07	4.66$\;\times \;10^{-9}$	4.97
	64	1.51$\;\times \;10^{-10}$	4.57	1.34$\;\times \;10^{-10}$	3.90	2.61$\;\times \;10^{-10}$	4.16
	128	7.50$\;\times \;10^{-12}$	4.33	8.78$\;\times \;10^{-12}$	3.93	1.55$\;\times \;10^{-11}$	4.08

**Table 5:** The temporal $L^{2}$ norm errors for case (b) with h=1/10, by modified schemes.

Scheme	*N*	α=0.1		α=0.5		α=0.9	
		$L^{2}$ Error	Rate	$L^{2}$ Error	Rate	$L^{2}$ Error	Rate
	16	6.60$\;\times \;10^{-4}$	-	5.23$\;\times \;10^{-4}$	-	3.32$\;\times \;10^{-4}$	-
BDF2 [1]	32	1.63$\;\times \;10^{-4}$	2.02	1.29$\;\times \;10^{-4}$	2.02	8.08$\;\times \;10^{-5}$	2.04
	64	4.06$\;\times \;10^{-5}$	2.01	3.20$\;\times \;10^{-5}$	2.01	2.00$\;\times \;10^{-5}$	2.02
	128	1.01$\;\times \;10^{-5}$	2.00	7.96$\;\times \;10^{-6}$	2.01	4.96$\;\times \;10^{-6}$	2.01
	16	5.07$\;\times \;10^{-5}$	-	4.43$\;\times \;10^{-5}$	-	4.43$\;\times \;10^{-5}$	-
BDF3 (25)	32	5.94$\;\times \;10^{-6}$	3.10	5.15$\;\times \;10^{-6}$	3.11	4.90$\;\times \;10^{-6}$	3.18
	64	7.20$\;\times \;10^{-7}$	3.04	6.22$\;\times \;10^{-7}$	3.05	5.76$\;\times \;10^{-7}$	3.09
	128	8.88$\;\times \;10^{-8}$	3.02	7.64$\;\times \;10^{-8}$	3.02	6.99$\;\times \;10^{-8}$	3.04
	16	6.38$\;\times \;10^{-5}$	-	5.24$\;\times \;10^{-5}$	-	4.62$\;\times \;10^{-5}$	-
BDF4 (28)	32	5.59$\;\times \;10^{-7}$	6.84	4.62$\;\times \;10^{-7}$	6.82	5.59$\;\times \;10^{-7}$	6.37
	64	3.15$\;\times \;10^{-8}$	4.15	2.61$\;\times \;10^{-8}$	4.15	3.11$\;\times \;10^{-8}$	4.17
	128	1.87$\;\times \;10^{-9}$	4.07	1.55$\;\times \;10^{-9}$	4.07	1.83$\;\times \;10^{-9}$	4.08

**Table 6:** The temporal $L^{2}$ norm errors for case (c) with h=1/10, by modified schemes.

Scheme	*N*	α=0.1		α=0.5		α=0.9	
		$L^{2}$ Error	Rate	$L^{2}$ Error	Rate	$L^{2}$ Error	Rate
	16	3.14$\;\times \;10^{-5}$	-	2.43$\;\times \;10^{-5}$	-	9.82$\;\times \;10^{-6}$	-
BDF2 [1]	32	7.82$\;\times \;10^{-6}$	2.01	6.04$\;\times \;10^{-6}$	2.01	2.51$\;\times \;10^{-6}$	1.97
	64	1.95$\;\times \;10^{-6}$	2.00	1.50$\;\times \;10^{-6}$	2.01	6.34$\;\times \;10^{-7}$	1.99
	128	4.86$\;\times \;10^{-7}$	2.00	3.75$\;\times \;10^{-7}$	2.00	1.59$\;\times \;10^{-7}$	1.99
	16	1.50$\;\times \;10^{-6}$	-	1.33$\;\times \;10^{-6}$	-	6.06$\;\times \;10^{-7}$	-
BDF3 (25)	32	1.94$\;\times \;10^{-7}$	2.95	1.68$\;\times \;10^{-7}$	2.99	6.91$\;\times \;10^{-8}$	3.13
	64	2.47$\;\times \;10^{-8}$	2.98	2.10$\;\times \;10^{-8}$	3.00	8.26$\;\times \;10^{-9}$	3.06
	128	3.11$\;\times \;10^{-9}$	2.99	2.63$\;\times \;10^{-9}$	3.00	1.01$\;\times \;10^{-9}$	3.03
	16	3.84$\;\times \;10^{-7}$	-	2.79$\;\times \;10^{-7}$	-	1.73$\;\times \;10^{-7}$	-
BDF4 (28)	32	1.08$\;\times \;10^{-8}$	5.15	8.20$\;\times \;10^{-9}$	5.09	6.16$\;\times \;10^{-9}$	4.81
	64	6.04$\;\times \;10^{-10}$	4.16	4.77$\;\times \;10^{-10}$	4.10	3.52$\;\times \;10^{-10}$	4.13
	128	3.57$\;\times \;10^{-11}$	4.08	2.88$\;\times \;10^{-11}$	4.05	2.11$\;\times \;10^{-11}$	4.06

## Data Availability

The data of numerical simulation used to support the findings of this study are included within the article.

## References

[1] Nong L., Chen A. (2021). Numerical schemes for the time-fractional mobile/immobile transport equation based on convolution quadrature. J. Appl. Math. Comput..

[2] Schumer R., Benson D.A., Meerschaert M.M., Baeumer B. (2003). Fractal mobile/immobile solute transport. Water Resour. Res..

[3] Hansen S.K., Berkowitz B. (2020). Modeling Non-Fickian Solute Transport Due to Mass Transfer and Physical Heterogeneity on Arbitrary Groundwater Velocity Fields. Water Resour. Res..

[4] Zhang Y., Zhou D., Yin M., Sun H., Wei W., Li S., Zheng C. (2020). Nonlocal transport models for capturing solute transport in one-dimensional sand columns: Model review, applicability, limitations and improvement. Hydrol. Process..

[5] Liu Q., Liu F., Turner I., Anh V., Gu Y.T. (2014). A RBF meshless approach for modeling a fractal mobile/immobile transport model. Appl. Math. Comput..

[6] Zhang Z., Liu X., Li X., Li X., Zhang X. (2020). A fast high-order compact difference method for the fractal mobile/immobile transport equation. Int. J. Comput. Math..

[7] Jiang H., Xu D., Qiu W., Zhou J. (2020). An ADI compact difference scheme for the two-dimensional semilinear time-fractional mobile-immobile equation. Comput. Appl. Math..

[8] Yin B., Liu Y., Li H. (2020). A class of shifted high-order numerical methods for the fractional mobile/immobile transport equations. Appl. Math. Comput..

[9] Nong L., Chen A., Yi Q., Li C. (2021). Fast Crank-Nicolson compact difference scheme for the two-dimensional time-fractional mobile/immobile transport equation. AIMS Math..

[10] Lubich C. (1988). Convolution quadrature and discretized operational calculus. I. BIT Numer. Math..

[11] Cuesta E., Lubich C., Palencia C. (2006). Convolution quadrature time discretization of fractional diffusion-wave equations. Math. Comput..

[12] Jin B., Li B., Zhou Z. (2017). An analysis of the Crank–Nicolson method for subdiffusion. IMA J. Numer. Anal..

[13] Jin B., Li B., Zhou Z. (2017). Correction of High-Order BDF Convolution Quadrature for Fractional Evolution Equations. SIAM J. Sci. Comput..

[14] Chen A., Nong L. (2020). Efficient Galerkin finite element methods for a time-fractional Cattaneo equation. Adv. Differ. Equ..

[15] Shi J., Chen M. (2020). Correction of High-Order BDF Convolution Quadrature for Fractional Feynman–Kac Equation with Lévy Flight. J. Sci. Comput..

[16] Nong L., Chen A., Cao J. (2021). Error estimates for a robust finite element method of two-term time-fractional diffusion-wave equation with nonsmooth data. Math. Model. Nat. Phenom..

[17] Chen A. (2021). Two efficient Galerkin finite element methods for the modified anomalous subdiffusion equation. Int. J. Comput. Math..

[18] Wang J., Wang J., Yin L. (2021). A Single-Step Correction Scheme of Crank–Nicolson Convolution Quadrature for the Subdiffusion Equation. J. Sci. Comput..

[19] Li C., Chen A. (2018). Numerical methods for fractional partial differential equations. Int. J. Comput. Methods Eng. Sci. Mech..

[20] Jin B., Lazarov R., Zhou Z. (2019). Numerical methods for time-fractional evolution equations with nonsmooth data: A concise overview. Comput. Methods Appl. Mech. Eng..

[21] Thomée V. (2006). Galerkin Finite Element Methods for Parabolic Problems.

